# Bioactive Polysaccharides from Fermented *Dendrobium officinale*: Structural Insights and Their Role in Skin Barrier Repair

**DOI:** 10.3390/molecules30132875

**Published:** 2025-07-06

**Authors:** Wanshuai Wang, Anqi Zou, Qingtao Yu, Zhe Wang, Daotong Tan, Kaiye Yang, Chao Cai, Guangli Yu

**Affiliations:** 1 Shandong Key Laboratory of Glycoscience and Glycotherapeutics, School of Medicine and Pharmacy, Ocean University of China, Qingdao 266003, China; wswang218@163.com (W.W.); 15610565165@163.com (A.Z.); wzhe0427@163.com (Z.W.); tandaotong123@163.com (D.T.); 2 Key Laboratory of Marine Drugs of Ministry of Education, School of Medicine and Pharmacy, Ocean University of China, Qingdao 266003, China; 3 Infinitus (China) Company Ltd., Guangzhou 510405, China; qingtao.yu@infinitus-int.com (Q.Y.); kyle.yang@infinitus-int.com (K.Y.); 4 Laboratory for Marine Drugs and Bioproducts, Qingdao Marine Science and Technology Center, Qingdao 266237, China

**Keywords:** fermented *Dendrobium officinale* polysaccharides, structure characterization, anti-inflammatory, antioxidant, skin barrier repair

## Abstract

*Dendrobium*, a prominent genus in the *Orchidaceae* family, has generated significant research attention due to its demonstrated biological potential, particularly its notable anti-inflammatory and antioxidant activities. In this study, two fractions of fermented *Dendrobium officinale* polysaccharides (FDOPs) were successfully isolated through a multi-stage purification strategy including gradient ethanol precipitation, gel column chromatography, and ion exchange chromatography with *Lactobacillus reuteri* CCFM863. Structural characterization revealed that both *Dendrobium officinale* polysaccharide fractions consisted of (1→4)-β-D-Man*p*, (1→4)-β-D-Glc*p*, and (1→4)-α-D-Glc*p* residues. The anti-inflammatory efficacy and keratinocyte-protective potential of FDOPs (FDOP-1A and FDOP-2A) were investigated by using lipopolysaccharide (LPS)-induced RAW264.7 and HaCaT cells models, which showed significant inhibitions on the inflammatory factors of monocyte chemoattractant protein-1 (MCP-1), tumor necrosis factor-alpha (TNF-α), nitric oxide (NO), and interleukin-1 beta (IL-1β); recovered levels of filaggrin (FLG), aquaporin 3 (AQP3), transient receptor potential vanilloid 4 (TRPV4), cathelicidin antimicrobial peptide (CAMP)/LL-37, and adiponectin (ADIPOQ); and the reduced protein expression of the TLR4/IκB-α/NF-κB/NLRP3 pathway. Notably, the FDOPs exhibited a remarkable reactive oxygen species (ROS) scavenging capacity, demonstrating superior antioxidant activity. Therefore, FDOPs show dual anti-inflammatory and antioxidant properties, making them suitable as active ingredients for modulating epidermal inflammation and promoting skin barrier repair.

## 1. Introduction

As the largest organ in the human body, the skin is of vital importance to maintain health. It serves as both a protective barrier against external insults and a sensory interface for environmental perception [1]. The skin is frequently affected by various factors, including environmental influences, genetic factors, and lifestyle choices, that may lead to inappropriate inflammatory responses that would significantly impair quality of life [2], such as long-term exposure to ultraviolet (UV) light. With the improvement in living standards and the growing demand for youthful skin, the treatment of skin barrier repair has attracted increasing attention [3]. Bioactive compounds derived from natural sources are emerging as promising therapeutic agents in dermatological treatment, particularly owing to their enhanced safety profiles and negligible systemic toxicity compared to conventional synthetic alternatives.

*Dendrobium*, a valuable traditional Chinese medicine belonging to the *Orchidaceae* family, is classified as a superior herb in the “Shennong Bencao Jing” due to its reputed benefits in alleviating paralysis, invigorating debility, and regulating “QI”, a key concept in traditional Chinese medicine that refers to the vital energy essential for maintaining physiological functions in the human body. A previous study on *Dendrobium* predominantly focused on its small molecule constituents, such as phenolics, alkaloids, and sesquiterpenes, with limited attention on its bioactive macromolecules [4,5,6,7]. *Dendrobium* is rich in a diverse array of components, including polysaccharides, flavonoids, alkaloids, pigments, and other low-molecular-weight entities [8,9,10]. Among these, *Dendrobium* polysaccharides are recognized as a principal bioactive component with 1,4-β-D-Man*p* and 1,4-β-D-Glc*p* structures, known for their antioxidant [11], anti-inflammatory [7], and anti-apoptotic activities [12,13], thereby holding significant potential in functional food applications [14]. Evidence indicates that *Dendrobium* polysaccharides play a crucial role in maintaining the structural and functional integrity of the skin barrier, referred to as “skin barrier integrity”, which may potentially enhance overall skin health [15].

Traditional *Dendrobium* polysaccharides typically exhibit the disadvantages of high molecular weight and complex structure. Microbial fermentation can transform natural polysaccharides into novel fermented products with enhanced biological activity, thereby improving their functional properties and expanding their application scope [16,17]. Utilizing the lactic acid probiotic *Bacillus* sp. DU-106 for the fermentation of natural polysaccharides can significantly increase the mannose content and enhance its immune-stimulating activity [18]. Fermentation with *Lactobacillus reuteri* CCFM8631 not only significantly increased the yield of fermented carbohydrates but also enhanced their skin care properties. The fermented *Dendrobium* polysaccharides exhibited better protection against SDS-induced injury in HaCaT cells, reduced lipopolysaccharide (LPS)-induced NO secretion in RAW 264.7 cells, and alleviated DNFB-triggered skin damage in a mouse model by mitigating inflammatory responses and promoting the restoration of normal skin barrier structure and function [19].

In this study, we established dermal bacterial infection-induced inflammation models using LPS-treated monocyte–macrophage RAW264.7 cells and keratinocyte HaCaT cells. To ascertain the impact of fractions of *Dendrobium officinale* polysaccharides (FDOPs) on epidermal barrier repair, we assessed the anti-inflammatory and antioxidant properties of FDOPs in LPS-induced RAW264.7 cells as well as their ability to promote the repair of skin barrier-related proteins in LPS-induced HaCaT cells.

## 2. Materials and Methods

### 2.1. Material

Trifluoroacetic acid (TFA), 1-phenyl-3-methyl-5-pyrazolone (PMP), phenol, sulphuric acid, alcohol, sodium borohydride (NaBH_4_), and methyl iodide (CH_3_I) were purchased from Sinopharm Chemical Reagent Co., Ltd. (Shanghai, China). Dimethyl sulfoxide (DMSO); DMSO-d6; and the monosaccharide standards mannose (Man), glucosamine (GlcN), rhamnose (Rha), glucuronic acid (GlcA), galacturonic acid (GalA), galactosamine (GalN), glucose (Glc), galactose (Gal), arabinose (Ara), and fucose (Fuc) were purchased from Sigma-Aldrich Co. (St. Louis, MO, USA). All other reagents used in this study were of analytical grade and purchased from commercial vendors, including the following: cell counting kit-8 (CCK8) assay kit (Beyotime, Shanghai, China, C0038), nitric oxide (NO) assay kit (Beyotime, S0021S), reactive oxygen species (ROS) assay kit (Beyotime, S00033S), monocyte chemoattractant protein-1 (MCP-1) ELISA kit (ABclonal, Woburn, MA, USA, RK00381), tumor necrosis factor-alpha (TNF-α) ELISA kit (ABclonal, RK00027), interleukin-1 beta (IL-1β) ELISA kit (ABclonal, RK00006), filaggrin (FLG) ELISA kit (ABclonal, RK09147), aquaporin 3 (AQP3) ELISA kit (ABclonal, RK00932), transient receptor potential vanilloid 4 (TRPV4) ELISA kit (abbexa, Cambridge, UK, abx383968), cathelicidin antimicrobial peptide (CAMP) ELISA kit (ABclonal, RK09135), adiponectin (ADIPOQ) ELISA kit (ABclonal, RK00060), Dulbecco’s modified eagle medium (DMEM) (Servicebio, Wuhan, China, G4511), fetal bovine serum (FBS) (ExCell Bio, Shanghai, China, FSP500), inhibitor of κB-α (IκB-α) rabbit mAb (Cell Signaling, Danvers, MA, USA, #4812), nuclear factor kappa-B (NF-κB) p65 rabbit mAb (Cell Signaling, #8242), cyclooxygenase-2 (COX-2) rabbit mAb (Cell Signaling, #122812), toll-like receptor 4 rabbit mAb (Cell Signaling, #38519), NOD-like receptor thermal protein domain associated protein 3 (NLRP3) rabbit mAb (Cell Signaling, #15101), and interleukin 18 (IL-18) rabbit mAb (Cell Signaling, #57058).

Fermented *Dendrobium officinale* (FDO) was provided by INFINITUS Company (Guangzhou, China). The fermentation process refers to a previous work [19]. *Lactobacillus reuteri* CCFM8631 was inoculated into a *Dendrobium officinale*-based medium at an initial concentration of 10^7^ CFU/mL. The fermentation was carried out at 37 °C and pH 6.0 for 16 h. During this process, the medium was continuously aerated to ensure sufficient oxygen supply for the growth and metabolism of the bacteria. The fermentation parameters, including temperature and pH, were strictly controlled to maintain optimal conditions for *Lactobacillus reuteri* CCFM8631. At the end of the fermentation, the cell counts were determined using the plate dilution method to evaluate the growth and viability of the strain.

### 2.2. Extraction of Polysaccharides

In this study, the dried FDO was first extracted using water (10:1 *v*/*w*) at 45 °C for 1 h, and then the supernatants were collected. Subsequently, the supernatants were precipitated with a final concentration of 40% ethanol (*v*/*v*) at 4 °C for 12 h. The supernatant obtained from the 40% ethanol solution was then collected and additional ethanol was added to reach a final ethanol concentration of 60% (*v*/*v*). These samples were then stored at 4 °C for 12 h and the precipitate was collected after centrifugation. The supernatant obtained from the 60% ethanol solution was then collected and additional ethanol was added to reach a final ethanol concentration of 80% (*v*/*v*). Again, these mixtures were stored at 4 °C for 12 h and the precipitate was obtained after centrifugation. The precipitate obtained after each alcohol precipitation was washed three times with acetone.

### 2.3. Isolation and Purification of Polysaccharides

The 60% alcoholic fraction (85 mg) was dissolved in deionized water (1 mL), and the solution was subjected to gel permeation chromatography through a Sephacryl S-400 column (Cytiva, Marlborough, MA, USA). The polysaccharide was eluted with 200 mL 0.3 M NH_4_HCO_3_ at a flow rate of 0.25 mL/min. The solution (1.5 mL per tube) was collected, and the polysaccharide component was isolated by repeatedly adding water until all ammonium bicarbonate was completely removed. FDOP-1A was dialyzed and lyophilized.

The 80% alcoholic fraction (155 mg) was dissolved in deionized water (10 mL), and the solution was subjected to a Q-Sepharose Fast Flow strong anion-exchange column. The polysaccharide was eluted with 600 mL distilled water at a flow rate of 3 mL/min. The solution (9 mL/tube) was collected, and the polysaccharide was determined using the phenol–sulfuric acid method. FDOP-2A was dialyzed and lyophilized.

### 2.4. Characterization of Polysaccharides

#### 2.4.1. Chemical Properties and Monosaccharide Composition Analysis

The phenol–sulfuric acid method was employed to determine the total carbohydrates using d-glucose as a standard sample [20]. The protein content was evaluated using a BCA assay.

The analysis of the monosaccharide composition was conducted according to the method described by Chen et al. [21] with slight modifications. Briefly, the polysaccharide sample was hydrolyzed with 4 M TFA at 110 °C for 6 h. Then, methanol was added and fully evaporated three times. The samples and monosaccharide standard mixture were mixed with a 0.3 M NaOH and PMP solution (dissolved in methanol) and incubated at 70 °C for 1 h. The reaction was stopped by neutralization with 0.3 M hydrochloric acid (HCl) and extracted with 0.5 mL of chloroform five times. The final aqueous layer was collected and analyzed using high-performance liquid chromatography (HPLC, Agilent1260, Agilent Technologies, Inc., Santa Clara, CA, USA). The monosaccharide standards mannose (Man), glucosamine (GlcN), rhamnose (Rha), glucuronic acid (GlcA), galacturonicacid (GalA), galactosamine (GalN), glucose (Glc), galactose (Gal), arabinose (Ara), and fucose (Fuc) were used to qualify the monosaccharide compositions.

#### 2.4.2. Molecular Weight Analysis

The molecular weight was measured via the method of performance of gel permeation chromatography–multi-high-angle laser light scattering–refractive index (HPGPC-MALLS-RI) described in our previously work [22]. The molecular weight distribution of the FDOPs was analyzed on an Agilent 1260 HPLC system and a DAWN HELEOS-II laser photometer (Wyatt Technology Co., Santa Barbara, CA, USA) equipped with both a Shodex Ohpak SB HQ 804 and 803 column (6 μm, 8.0 × 300 mm, Showa Denko, Japan) and eluted with a 0.1 M NaNO_3_ solution at 35 °C with a flow rate of 0.6 mL/min. The data acquisition and analysis were performed using Astra software (version 6.1.1).

#### 2.4.3. Methylation Analysis of FDOPs

According to the method of Anumula [23], different residues with slightly modified linkage types were determined via a methylation analysis. Briefly, the polysaccharide samples were dissolved in DMSO and methylated with sodium hydride (NaH) and methyl iodide (CH_3_I) for 3 h. The reaction was terminated with water and extracted three times with chloroform. After methylation, the chloroform layers were concentrated under reduced pressure and analyzed via infrared (IR) spectroscopy. The disappearance of the broad O-H stretching vibration at approximately 3385 cm^−1^ indicated that the full methylation was complete, and if not, the full methylation steps were repeated. The completely methylated product was added to 2 M TFA and degraded at 110 °C for 4 h and then reduced with 0.1 M NaOH (containing 0.48 M NaBD_4_) at room temperature for 4 h. The reduced sample was added to anhydrous pyridine and acetic anhydride for acetylation. The resulting partially methylated alditol acetates (PMAAs) were then determined by gas chromatography–mass spectrometry (GC-MS), and the data analysis employed the CCRC database.

#### 2.4.4. FT-IR Spectroscopy Analyses

The polysaccharide samples (1–2 mg) were mixed with 50 mg dried KBr and pressed into a transparent pellet. Then, the spectrum was recorded by using a KBr pellet method on an FT-IR spectrometer (Nexus 470, Nicolet, Madison, WI, USA) in the region of 400–4000 cm^−1^. The data acquisition and analysis were performed using OMNIC software (version 8.2).

#### 2.4.5. NMR Analyses

The polysaccharide samples were dissolved in D_2_O (99.96%, Sigma-Aldrich, Inc., St. Louis, MO, USA), subjected to freeze-drying, and exchanged with D_2_O three times. Finally, these samples were dissolved in the D_2_O solution at a concentration of 20 mg/mL, and were then subjected to the acquisition of their NMR spectra. The ^1^H, ^13^C, and ^1^H-^13-^C HSQC spectra were obtained using an Agilent DD2 500 MHz spectrometer (Agilent, USA).

### 2.5. Evaluation of FDOPs Skin Care Effects In Vivo

#### 2.5.1. Cell Culture

HaCaT cells and RAW 264.7 cells were purchased from the Chinese Type Culture Collection Center (Wuhan, China). The cells were cultured in DMEM containing 10% FBS and incubated at 37 °C with 5% CO_2_. Prior to use, the polysaccharide samples were dissolved in PBS and sterilized using a 0.22 μm membrane filter.

#### 2.5.2. Cell Viability Assay

LPS was used to induce cellular inflammatory responses, simulating the inflammatory environment following skin infection or injury. HaCaT cells in a logarithmic phase (3 × 10^4^ cells/well) were seeded into 96-well plates and divided into three groups: (1) control (CON) group (medium only), (2) model group (LPS), and (3) treatment group (FDO/FDOP-1A/FDOP-2A + LPS). The cells were exposed to LPS (5 μg/mL) for 24 h. Subsequently, the medium was replaced with fresh DMEM containing 100 μg/mL of the samples (FDO, FDOP-1A, FDOP-2A) and cultured for an additional 24 h. Cell survival rates were determined using the CCK-8 assay.

#### 2.5.3. NO Assay

RAW 264.7 cells were seeded in 96-well plates (3 × 10^4^ cells/well). After 24 h, the cells were pretreated by the samples (100 μg/mL) for 2 h, followed by stimulation with LPS (1 μg/mL) for 24 h. Finally, the content of NO in the supernatant was tested using an NO assay kit according to the manufacturer’s instructions.

#### 2.5.4. ELISA Assay

HaCaT cells were seeded in 6-well plates (1 × 10^6^ cells/well). After 12 h, the cells were pretreated by the samples (100 μg/mL) for 24 h, followed by stimulation with LPS (5 μg/mL) for 24 h. The content of MCP-1, TNF-α, IL-1β, FLG, AQP3, and TRPV4 in the supernatant was evaluated using the ELISA kit according to the manufacturer’s instructions.

#### 2.5.5. Western Blotting Analysis

RAW 264.7 cells were seeded in 6-well plates (1 × 10^6^ cells/well). After 24 h, the cells were pretreated by the samples (100 μg/mL) for 2 h. The cells were then stimulated with LPS (1 μg/mL) for 24 h. Protein samples were collected using cell signaling technology (Boston, MA, USA). The protein content was determined by using a BCA protein kit (KeyGen, Nanjing, Jiangsu, China). Equal amounts of the proteins were loaded into SDS-PAGE gels and imprinted on polyvinylidene fluoride membranes (Bio-Rad, Hercules, CA, USA).

#### 2.5.6. ROS Assay

RAW 264.7 cells were seeded in 6-well plates (1 × 10^6^ cells/well). After 24 h, the cells were pretreated by the samples (100 μg/mL) for 2 h, followed by stimulation with LPS (1 μg/mL) for 24 h. The ROS levels were determined using an ROS detection kit, following the manufacturer’s instructions, and fluorescence intensity was measured using flow cytometry and laser confocal microscopy.

### 2.6. Statistical Analysis

All data were collected from three independent experimental replicates. Inter-group comparisons were performed using one-way ANOVA followed by Tukey’s post hoc test using GraphPad Prism 9.0 software. The data are presented as the mean ± SD of three independent experiments. Statistically significant differences were defined as * *p* < 0.05, ** *p* < 0.01, *** *p* < 0.001, and **** *p* < 0.0001.

## 3. Results

### 3.1. Purification and Chemical Properties of FDOPs

**Figure 1:**
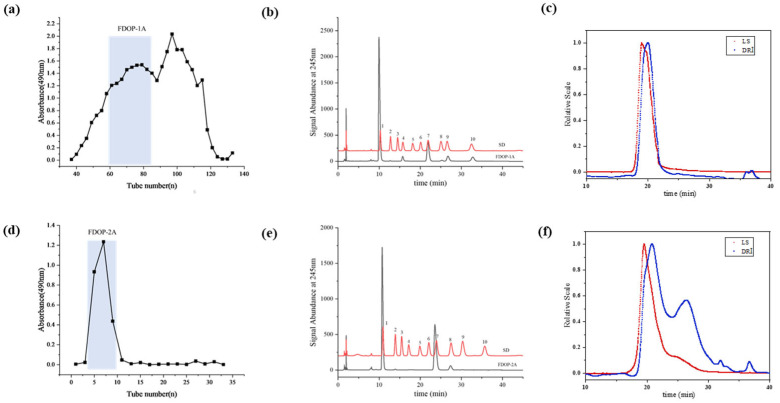
The purification and chemical properties of FDOP-1A and FDOP-2A: (**a**) A stepwise elution curve of the crude polysaccharide (60% alcoholic fraction) on a Sephacryl S-400 column. (**b**) The monosaccharide composition of FDOP-1A (1. Man, 2. GlcN, 3. Rha, 4. GlcA, 5. GalA, 6. GalN, 7. Glc, 8. Gal, 9. Ara, and 10. Fuc). (**c**) The molecular weight distribution by HPGPC-RI-MALLS of FDOP-1A. (**d**) A stepwise elution curve of the crude polysaccharide (80% alcoholic fraction) on a Q-Sepharose Fast Flow (QFF) strong anion-exchange column. (**e**) The monosaccharide composition of FDOP-2A. (**f**) The molecular weight distribution by HPGPC-RI-MALLS of FDOP-2A.

The 60% and 80% alcoholic fractions were obtained by ethanol precipitation according to the modified and optimized extraction conditions [24]. As shown in Figure 1a, the 60% alcoholic fraction was purified through sequential elution using a Sephacryl S-400 column. The first fraction (FDOP-1A) was collected using 0.3 M NH_4_HCO_3_ as the solution. Subsequently, the 80% alcoholic fraction was purified using a Q-Sepharose Fast Flow strong anion-exchange column (Figure 1d), yielding the polysaccharide fraction (FDOP-2A). The purified fractions FDOP-1A and FDOP-2A were then utilized for subsequent experiments.

The phenol–sulfuric acid analysis revealed that the total carbohydrate contents of FDOP-1A and FDOP-2A were 96.05% and 100.00%, respectively. Moreover, the protein contents of FDOP-1A and FDOP-2A were determined to be 2.58% and 0.00%, respectively. The monosaccharide composition of FDOP-1A was determined via high-performance liquid chromatography (HPLC) with pre-column derivatization, and PMP is a commonly used ultraviolet absorption reagent to improve the detection sensitivity of the released monosaccharides. As shown in Figure 1b, FDOP-1A is a heteropolysaccharide composed of mannose, glucose, galactose, glucuronic acid, fucose, and arabinose at a molar ratio of 73.25:10.53:0.91:5.9:8.46:0.96. FDOP-2A is a heteropolysaccharide consisting of mannose and glucose at a molar ratio of 66.73:33.27 (Figure 1e).

The molecular weight distribution of the FDOPs is presented in Figure 1. The results indicate that FDOP-1A has a molecular weight of approximately 184.2 kDa (Figure 1c), while FDOP-2A exhibits two distinct molecular weight fractions, at approximately 39.36 kDa and 7.89 kDa (Figure 1f).

### 3.2. Structural Characterization of FDOPs

#### 3.2.1. FT-IR Spectrum Analysis

**Figure 2:**
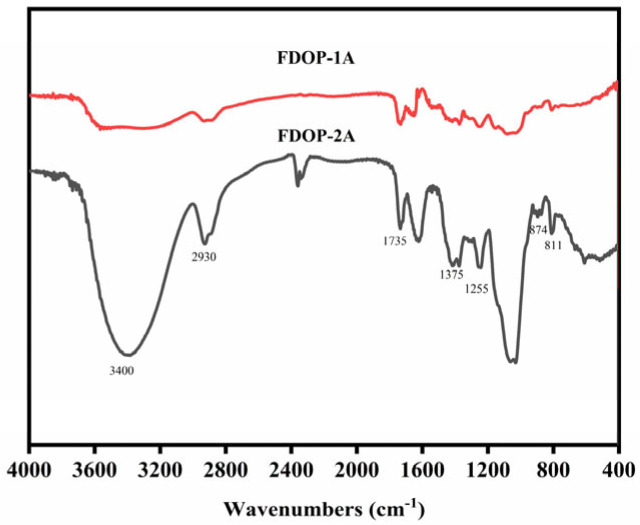
FT-IR spectra of FDOP-1A and FDOP-2A.

As shown in Figure 2, the strong broad characteristic peak at approximately 3400 cm^−1^ is attributed to the O-H stretching vibration of pyranose rings. The band at approximately 2935 cm^−1^ corresponds to the C-H stretching vibration [25]. In addition, the peaks at around 1735, 1375, and 1255 cm^−1^ are assigned to the valence vibration of C=O, the symmetric C-H bending vibration of the methyl groups, and the C-O vibration of the *O*-acetyl groups, respectively [26,27]. The peaks at 874 and 811 cm^−1^ represent the typical absorption bands of β-configurations and α-configurations, respectively [28,29]. The FT-IR results indicate that FDOP-1A and FDOP-2A are typical α- and β-configuration polysaccharides with *O*-acetyl substitutions.

#### 3.2.2. Methylation Analysis

Methylation analysis is a widely used technique in the structural analysis of complex polysaccharides, providing insights into the types and linkages of glycosyl units.

The methylation analysis results indicated that FDOP-1A and FDOP-2A had similar sugar residues. As shown in Table 1 and Figures S1 and S2, four alditol acetates, namely 2,3,4,6-Me4-Man*p*, 2,3,4,6-Me4-Glc*p*, 2,3,6-Me3-Glc*p*, and 2,3,6-Me3-Man*p*, were clearly detected by GC–MS. Therefore, four corresponding glycosidic linkages were identified as T-linked-Man*p*, T-linked-Glc*p*, 1,4-linked-Glc*p*, and 1,4-linked-Man*p*, respectively. Based on the relative molar ratio of individual alditol acetates and the monosaccharide analyses, it can be inferred that both FDOP-1A and FDOP-2A are mainly composed of 1,4-linked-Glc*p* and 1,4-linked-Man*p*. Methylation analysis revealed that the ratios between (1→4)-β-D-Man*p* and (1→4)-α-D-Glc*p* were determined to be 1.87:1 and 1.57:1 in FDOP-1A and FDOP-2A, respectively. Based on the molecular weight determinations, the degree of polymerization (DP) was calculated to be 1137 for FDOP-1A, while FDOP-2A displayed a DP distribution ranging from 170 to 243.

#### 3.2.3. NMR Analysis

**Figure 3:**
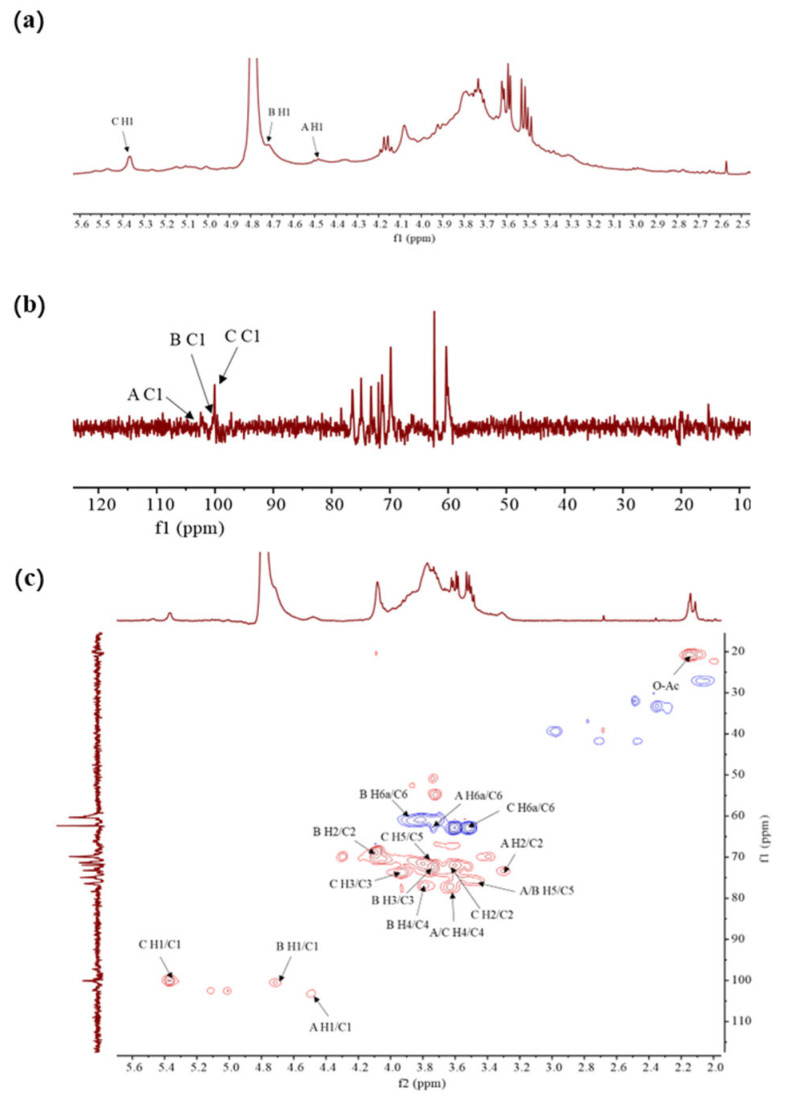
The NMR spectra of FDOP-1A. (**a**) ^1^H-NMR spectrum of FDOP-1A in D_2_O; (**b**) ^13^C-NMR spectrum of FDOP-1A; and (**c**) ^1^H-^13^C HSQC spectrum of FDOP-1A.

The structural characteristics of FDOP-1A and FDOP-2A were verified using the results of the NMR analysis, including ^1^H-NMR, ^13^C-NMR, and ^1^H-^13^C HSQC experiments. The ^1^H-NMR spectrum of FDOP-1A (Figure 3a) revealed that the anomeric proton signals were concentrated in the range of 3.5–5.5 ppm, which is a typical signal for polysaccharides [30].

The ^1^H-NMR spectrum of FDOP-1A revealed three major anomeric proton signals in the range of 4.4–5.5 ppm, indicating three types of monosaccharides. The strong signal of 4.79 ppm corresponds to the solvent D_2_O, while the peaks at 4.49, 4.72, and 5.38 ppm were designated as residues A, B, and C, respectively. The chemical shift at 5.38 ppm (residue C) was assigned to an α-linked glycosidic residue, whereas the signals at 4.49 ppm (residue A) and 4.72 ppm (residue B) were ascribed to β-anomeric protons [31]. These findings are consistent with the FT-IR analysis results. The ^13^C- NMR (Figure 3b) and ^1^H-^13^C HSQC spectra of FDOP-1A (Figure 3c) further confirmed the presence of three anomeric carbon signals. According to the previous literature’s data [29], the signal peaks in the range of 170–180 ppm and 20–21 ppm were attributed to carbonyl and methyl groups, respectively. Additionally, the lowest field signal at 5.47 ppm was assigned to the H-2 of 2-*O*-acetyl-(1→4)-β-D-Man*p* in the ^1^H-NMR spectrum [32]. Three anomeric carbon signals were observed in the chemical shift range of 97–103 ppm, corresponding to residues A, B, and C at 100.03 ppm, 100.53 ppm, and 103.30 ppm, respectively.

For residue A, the anomeric signal at 4.49 ppm suggests that residue A is likely a β-configuration unit. The HSQC spectrum assigned the carbon chemical shifts as follows: C-1 (103.52 ppm), C-2 (73.23 ppm), C-3 (75.06 ppm), C-4 (78.37 ppm), C-5 (74.94 ppm), and C-6 (62.36 ppm). Based on the NMR analysis, methylation analysis, and previous literature data, it can be deduced that residue A corresponds to (1→4)-β-D-Glc*p*. Similarly, residues B and C can be inferred as (1→4)-β-D-Man*p* and (1→4)-α-D-Glc*p*, respectively. All these results are in agreement with the previous literature’s data [31].

The NMR results of FDOP-2A (Figure S3) were similar to those of FDOP-1A. It can therefore be inferred that FDOP-2A also contains similar glycosidic bonds as FDOP-1A, namely (1→4)-β-D-Glc*p*, (1→4)-β-D-Man*p*, and (1→4)-α-D-Glc*p*, respectively.

### 3.3. FDOPs Inhibit LPS-Induced Inflammatory Cytokine Release in RAW264.7 Cells

In our pre-experiment, the inhibitory effect of FDOP-1A on MCP-1 was evaluated at concentrations of 50 μg/mL, 100 μg/mL, and 200 μg/mL, respectively. The results showed that the inhibitory effect was stronger at 100 μg/mL (Figure S4). For a more effective comparison, the concentration of the sample was set at 100 μg/mL for the subsequent experiment.

**Figure 4:**
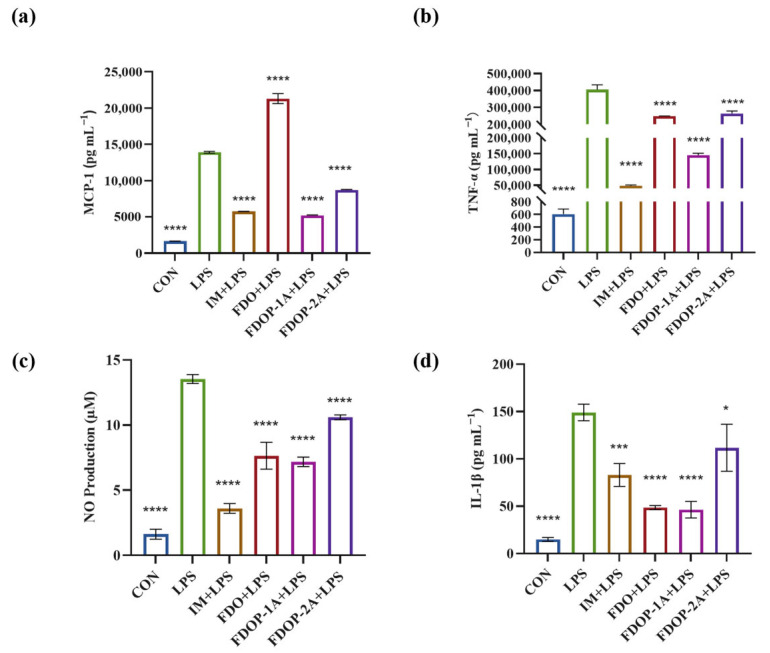
Effects of FDOP-1A and FDOP-2A on the production of MCP-1, TNF-α, NO, and IL-1β in LPS-induced RAW 264.7 cells: (**a**) MCP-1 content, (**b**) TNF-α content, (**c**) NO production, and (**d**) IL-1β content. * *p* < 0.05, *** *p* < 0.001, and **** *p* < 0.0001.

To evaluate the anti-inflammatory effects of FDO, FDOP-1A, and FDOP-2A, Indomethacin (IM) was used as a positive control group. The release of inflammatory factors MCP-1, TNF-α, NO, and IL-1β was detected. The results indicated that pretreatment with FDOP-1A and FDOP-2A effectively inhibited the release of MCP-1, TNF-α, NO, and IL-1β (Figure 4a–d) to varying degrees, demonstrating their effective anti-inflammatory activity. In addition, FDOPA-1A demonstrated a more pronounced inhibitory effect.

### 3.4. FDOPs Scavenge Intracellular ROS in RAW264.7 Cells

**Figure 5:**
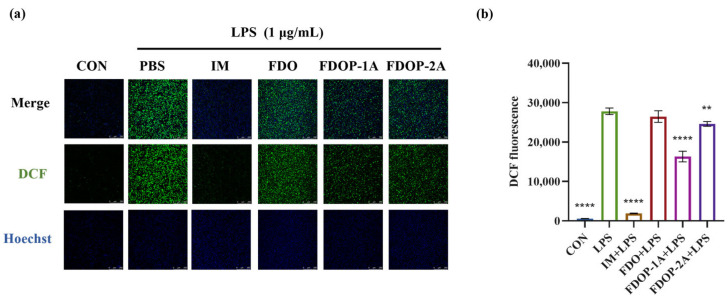
Effects of FDOP-1A and FDOP-2A on ROS levels: (**a**) DCF fluorescent probe was used to stain intracellular ROS in cells. (**b**) Relative DCF fluorescence intensity. ** *p* < 0.01, and **** *p* < 0.0001.

2′,7′-Dichlorofluorescein (DCF) was employed to label ROS. The fluorescence intensity was detected via laser confocal microscopy and flow cytometry. The results showed that the fluorescence intensities of both the FDOP-1A and FDOP-2A groups were lower than that of the LPS group (Figure 5a,b). The experimental results confirmed that FDOP-1A and FDOP-2A exhibited scavenging and inhibitory effects on intracellular ROS, with FDOP-1A demonstrating a stronger capacity to clear ROS compared to FDOP-2A.

### 3.5. FDOPs Inhibit TLR4/NF-κB/NLRP3 Pathway for Anti-Inflammatory Activity

**Figure 6:**
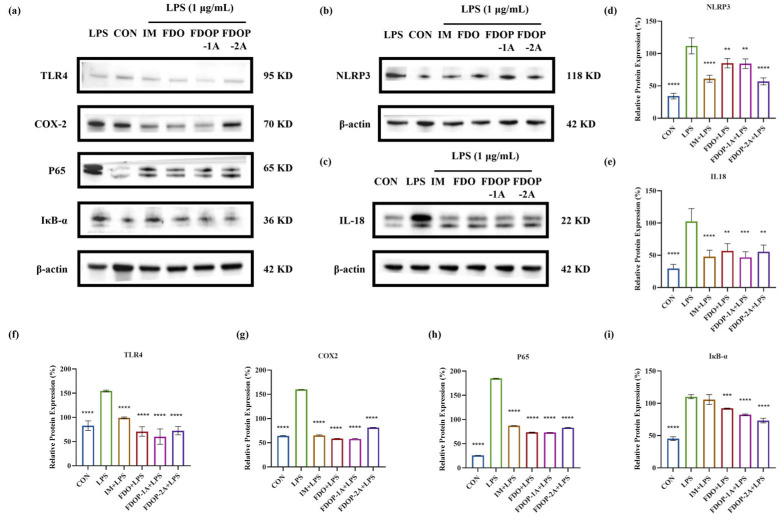
The relative protein expression of NLRP3, IL-18, TLR4, COX-2, P65, and IκB-α in LPS-induced RAW 264.7 cells: (**a**–**c**) The expression of NLRP3, IL-18, TLR4, COX-2, P65, and IκB-α detected by Western blot. (**d**–**i**) Statistical analyses on NLRP3, IL-18, TLR4, COX-2, P65, and IκB-α. ** *p* < 0.01, *** *p* < 0.001, and **** *p* < 0.0001.

The expression levels of NLRP3, IL-18, TLR4, COX-2, P65, and IκB-α in LPS-induced RAW 264.7 cells were detected through Western blot to further determine whether the anti-inflammatory effect of FDOP-1A and FDOP-2A was related to the inhibition of inflammatory pathways (Figure 6a–c). The expression levels of these proteins were significantly increased in the LPS group compared to those in the control group. In contrast, FDOP-1A and FDOP-2A resulted in reduced expression levels of NLRP3, IL-18, TLR4, COX-2, P65, and IκB-α. The detailed findings are presented in Figure 6d–i. The above results prove that FDOP-1A and FDOP-2A can alleviate the inflammatory response by inhibiting the TLR4/IκB-α/NF-κB signaling pathway in RAW264.7 cells, thereby regulating the release of inflammatory cytokines such as TNF-α and MCP-1.

### 3.6. FDOPs Attenuate LPS-Induced Damage in HaCaT Cells

#### 3.6.1. Cell Viability

**Figure 7:**
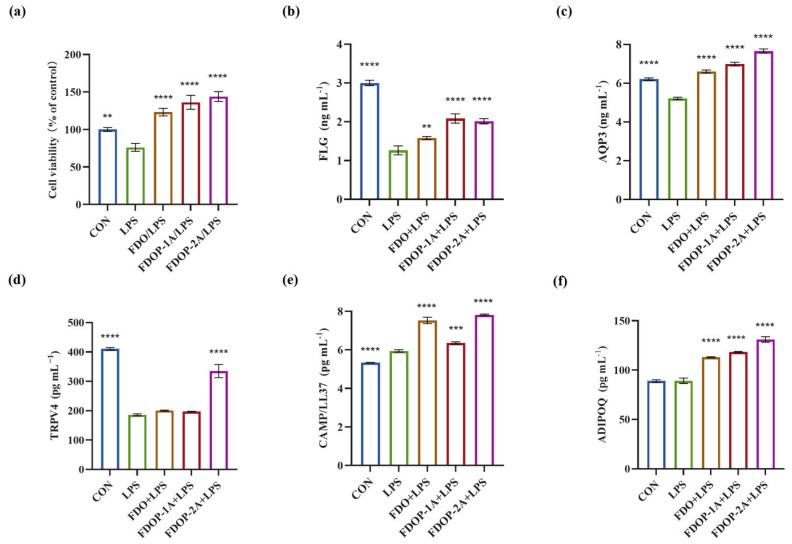
Effects of FDOP-1A and FDOP-2A in LPS-induced HaCaT cells: (**a**) cell viability; (**b**) FLG content; (**c**) AQP3 content; (**d**) TRPV4 content; (**e**) CAMP/LL37 production; and (**f**) ADIPOQ production. ** *p* < 0.01, *** *p* < 0.001, and **** *p* < 0.0001.

To determine whether the FDOPs exert ameliorative effects on LPS-induced HaCaT cells, we assessed their cell viability. A significant increase in HaCaT cells was observed when the cells were pre-incubated with 100 μg/mL FDO, FDOP-1A, or FDOP-2A. As depicted in Figure 7a, following a 24 h treatment with 5 μg/mL LPS, the cell viability of the HaCaT cells was reduced to approximately 76.09%. Upon replacement with fresh media containing FDO, FDOP-1A, and FDOP-2A, cell viability significantly increased, reaching 1.62-, 1.79-, and 1.89-fold that of the LPS group, respectively.

#### 3.6.2. FDOPs Enhance the Skin Barrier-Related Proteins in HaCaT Cells

The effects of FDOP-1A and FDOP-2A on skin barrier function and inflammation were subsequently investigated in this study. FLG, a crucial protein for skin barrier integrity, was significantly reduced in the LPS group, but its levels were restored after FDOP-1A and FDOP-2A treatment (Figure 7b). AQP3, which regulates skin hydration, was also elevated by FDOP-1A and FDOP-2A compared to the LPS group (Figure 7c). TRPV4, involved in skin water and electrolyte balance, showed a significant increase only in the FDOP-2A group (Figure 7d). CAMP/LL-37, an anti-inflammatory peptide, was upregulated by FDOP-1A and FDOP-2A, potentially enhancing innate immunity (Figure 7e). ADIPOQ, an anti-inflammatory hormone-like protein, was significantly increased by FDOP-1A and FDOP-2A compared to the LPS group (Figure 7f). Overall, FDOP-1A and FDOP-2A improved skin barrier function and hydration, with FDOP-2A demonstrating stronger antioxidant activity, potentially due to its smaller molecular weight.

## 4. Discussion

The *Dendrobium* genus has garnered significant attention due to its biological potential. Recent studies have shown that polysaccharides are one of the main bioactive components of *Dendrobium*, possessing multiple biological activities such as antioxidant, immunomodulatory, hepatoprotective, anti-tumor, and anti-inflammatory activities [33,34]. Ge et al. [35] obtained a polysaccharide from artificially cultivated *Dendrobium huoshanense*, which was composed of mannose and glucose at a molar ratio of 1.89:1.00, with 1, 4-Man*p* and 1, 4-Glc*p* bonds as its main chains. This polysaccharide could reduce the infiltration of inflammatory cells and inhibit the increase in TNF-α and IL-1β levels in serum and lung tissue induced by cigarette smoke, and its anti-inflammatory effect was mediated by regulating the NF-κB and MAPK signaling pathways. Fan et al. [36] isolated six polysaccharides from *Dendrobium officinale* using a sequential alcohol precipitation technique. All six fractions exhibited potent antioxidant properties in vitro, with lower molecular weight fractions demonstrating higher antioxidant activity.

Our study aimed to explore the anti-inflammatory and antioxidant properties of polysaccharides isolated from FDO. Two polysaccharide fractions, FDOP-1A and FDOP-2A, were successfully obtained through a multistage purification strategy, including gradient ethanol precipitation, gel column chromatography, and ion exchange chromatography. Their structural properties were determined to possess (1→4)-β-D-Man*p*, (1→4)-β-D-Glc*p*, and (1→4)-α-D-Glc*p* glycosidic linkages with distinct molecular weights (FDOP-1A: ~184.2 kDa; FDOP-2A: ~39.36/7.89 kDa) by NMR, FT-IR, methylation, and other techniques. This is consistent with previous studies that have shown similar structural features in polysaccharides from *Dendrobium* species [34]. Chu et al. [37] demonstrated that the ultrasonic treatment of *Dendrobium officinale* polysaccharides enhances their antioxidant activity and anti-inflammatory effects. Specifically, medium-power ultrasonic-treated *Dendrobium officinale* polysaccharides showed the highest scavenging activity against ROS and RNS radicals and upregulated anti-inflammatory/antioxidant proteins (Nrf2, HO-1, NQO1) via the Nrf2/HO-1/NQO1 pathway. Building on the findings from other studies on *Dendrobium* polysaccharides, our subsequent research aimed to further investigate the role of these bioactive compounds in skin barrier repair, with a particular emphasis on their anti-inflammatory and antioxidant properties.

The activity of polysaccharides can be influenced by a variety of factors, including molecular weight, monosaccharide composition, and the conformation of the glycosidic bonds [38]. Studies on *Xylaria nigripes* polysaccharides revealed that higher molecular weight fractions exhibit enhanced anti-inflammatory activity. A fraction with 853 kDa demonstrated significantly greater efficacy than their lower-molecular-weight counterparts [39]. These experiments show a similar trend to our results in this study (Figure 4), where FDOP-1A (184.2 kDa) showed superior anti-inflammatory effects compared to FDOP-2A (39.36/7.89 kDa). On the other hand, it has been previously reported that the mannose content in *Dendrobium* polysaccharides is positively correlated with its antioxidant activity [33]. Huang et al. [40] isolated two mannose-rich polysaccharides from *Dendrobium officinale*. These polysaccharides exhibited mild immune stimulation and antioxidant activity, among which the higher mannose content showed better antioxidant activity. In our study, the mannose content of FDOP-1A was higher than that of FDOP-2A, which might be another factor contributing to the difference in antioxidant activity observed.

**Figure 8:**
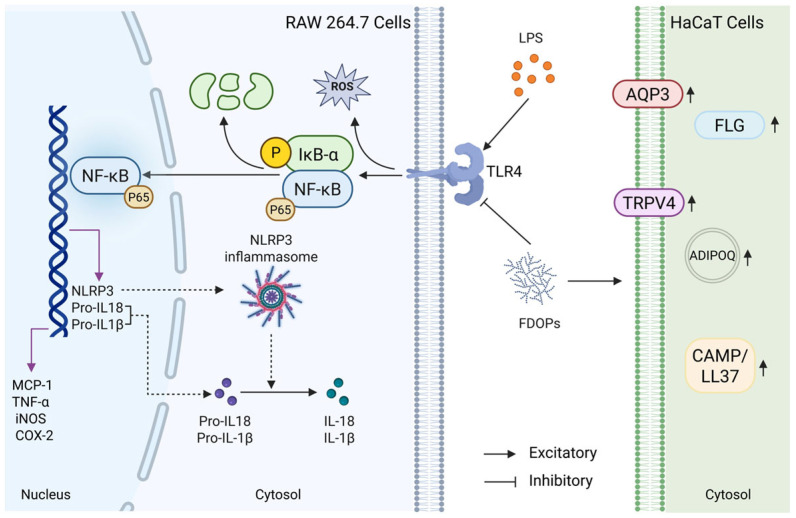
Summary of the anti-inflammatory and antioxidant mechanisms of FDOPs in vitro.

Macrophages, as critical effector cells of the innate immune system, play a crucial role in protecting the host from infections and tissue damage through functions such as phagocytosis, cytokine secretion, and antigen presentation [41]. Keratinocytes constitute approximately 95% of the cellular mass in the human epidermis and play a pivotal role in various skin conditions [42]. For instance, the immortal human keratinocyte line HaCaT is often used in the related research of skin lesions in vitro [43]. LPS, a major component of the outer membrane of Gram-negative bacteria, is one of the most common inflammatory stimulants [44]. Therefore, LPS-induced RAW264.7 and HaCaT cell models were used to evaluate the anti-inflammatory efficacy and keratinocyte protective potential of FDOP-1A and FDOP-2A. In this study, it was demonstrated that FDOP-1A and FDOP-2A suppressed the levels of MCP-1, TNF-α, NO, and IL-1β in LPS-induced RAW264.7 macrophages and recovered the levels of skin barrier-related proteins such as FLG, AQP3, and TRPV4 in HaCaT cells (Figure 8). In previous studies [45,46], most of the research on signaling pathways focused on TLR4/NF-κB, lacking more detailed studies and ignoring the roles of NLRP3 and IκB-α in these pathways. Through Western blotting and Elisa, it was discovered that FDOP-1A and FDOP-2A reduced the expression levels of proteins in the TLR4/IκB-α/NF-κB/NLRP3 signaling pathway, indicating the involvement of this pathway in the anti-inflammatory effects of FDOP-1A and FDOP-2A. These findings are consistent with previous studies that have shown the anti-inflammatory properties of polysaccharides from *Dendrobium* [47,48,49].

In view of the above findings, this study aims to further explore the potential applications of fermented *Dendrobium officinale* polysaccharides, especially in the repair of the skin barrier. By taking advantage of their established antioxidant and anti-inflammatory properties, we hypothesize that these polysaccharides may offer significant benefits in promoting skin health and alleviating diseases related to damaged skin barriers. This study enhances the understanding of the mechanism underlying the biological activity of fermented *Dendrobium officinale* polysaccharides and evaluates their potential as therapeutic agents for skin-related diseases.

## 5. Conclusions

This study successfully isolated and purified two polysaccharides, FDOP-1A and FDOP-2A, from FDO using gradient ethanol precipitation and chromatographic techniques. Structural characterization revealed that both fractions primarily consisted of (1→4)-β-D-Man*p*, (1→4)-β-D-Glc*p*, and (1→4)-α-D-Glc*p*, with distinct molecular weights (FDOP-1A: ~184.2 kDa; FDOP-2A: ~39.36/7.89 kDa) and monosaccharide compositions. FDOP-1A exhibited a higher mannose content, while FDOP-2A demonstrated a higher ratio of glucose. FDOP-1A and FDOP-2A possessed potent anti-inflammatory and antioxidant properties, demonstrated by the in vitro evaluations in this study. Both fractions significantly suppressed LPS-induced inflammatory cytokine release (MCP-1, TNF-α, NO, and IL-1β) in RAW264.7 cells by inhibiting the TLR4/IκB-α/NF-κB/NLRP3 signaling pathway. Notably, FDOP-1A outperformed FDOP-2A in suppressing ROS production and inflammatory mediators, attributed to its higher molecular weight and structural complexity. Conversely, FDOP-2A exhibited superior efficacy in enhancing the skin barrier-related proteins (FLG, AQP3, TRPV4, CAMP, ADIPOQ) in HaCaT cells, likely due to its smaller molecular size and glucose-rich composition. The dual anti-inflammatory and antioxidant properties of FDOP-1A and FDOP-2A, together with their demonstrated ability to promote epidermal barrier repair, indicate that these compounds hold significant potential for further exploration. The observed bioactivities provide a foundation for investigating their application prospects as natural components in therapeutic strategies targeting skin inflammation, oxidative stress, and barrier impairment. Future studies should focus on in vivo validation and clinical translation in the application of dermatology.

## Figures and Tables

**Table 1:** Major glycosidic linkage analysis of FDOP-1A and FDOP-2A.

Methylated Sugars	Linkage Types	Molar Ratio (%)		Mass Fragment (*m*/*z*)
		FDOP-1A	FDOP-2A	
2,3,4,6-Me4-Man*p*	T-linked-Man*p*	-	9.89	59, 71, 87, 102, 113, 118, 129, 145, 162, 174, 189, 205
2,3,4,6-Me4-Glc*p*	T-linked-Glc*p*	5.65	-	59, 71, 87, 102, 118, 129, 145, 162, 175, 205
2,3,6-Me3-Glc*p*	1,4-linked-Glc*p*	19.19	28.29	59, 71, 73, 87, 99, 102, 113, 118, 129, 131, 162, 173, 233
2,3,6-Me3-Man*p*	1,4-linked-Man*p*	37.13	44.43	59, 71, 87, 99, 102, 113, 118, 129, 142, 162, 173, 233

## Data Availability

The original contributions presented in this study are included in the article/supplementary material. Further inquiries can be directed to the corresponding authors.

## Supplementary Materials

The following supporting information can be downloaded at https://www.mdpi.com/article/10.3390/molecules30132875/s1, Figure S1: The GC-MS data of the partially methylated alditol acetates of FDOP-1A. (a) GC-MS elution spectra of FDOP-1A after methylation. (b) Mass spectrometry fragments of Man*p*-(1-. (c) Mass spectrometry fragments of -4)-Man*p*-(1-. (d) Mass spectrometry fragments of -4)-Glc*p*-(1-; Figure S2: The GC-MS data of the partially methylated alditol acetates of FDOP-2A. (a) GC-MS elution spectra of FDOP-2A after methylation. (b) Mass spectrometry fragments of Man*p*-(1-. (c) Mass spectrometry fragments of -4)-Man*p*-(1-. (d) Mass spectrometry fragments of -4)-Glc*p*-(1-; Figure S3: The NMR spectra of FDOP-2A: (a) ^1^H-NMR spectrum of FDOP-2A in D_2_O at 60 °C; (b) ^13^C-NMR spectrum of FDOP-2A; (c) ^1^H-^13^C HSQC spectrum spectra of FDOP-2A; Figure S4. The effect of FDOP-1A on the production of MCP-1 in LPS-induced RAW 264.7 cells at concentrations of 50 μg/mL, 100 μg/mL, and 200 μg/mL.
