# Supplementary material for: Bioactive Polysaccharides from Fermented Dendrobium officinale: Structural Insights and Their Role in Skin Barrier Repair
Source: Molecules. 2025 Jul 6;30(13):2875. doi: 10.3390/molecules30132875 (PMC12250875; doi:10.3390/molecules30132875)
Supplement: Supplementary file 1 [file molecules-30-02875-s001.zip › molecules-3682562-supplementary.pdf]

Bioactive Polysaccharides from Fermented *Dendrobium officinale*: Structural Insights and Their Role in Skin Barrier Repair

Wanshuai Wang [1,2,†], Anqi Zou [1,2,†], Qingtao Yu [3,†], Zhe Wang [1,2], Daotong Tan [1,2], Kaiye Yang [3], Chao Cai [1,2,4,*] and Guangli Yu [1,2,4,*]

1. Shandong Key Laboratory of Glycoscience and Glycotherapeutics, School of Medicine and Pharmacy, Ocean University of China, Qingdao 266003, China; wswang218@163.com (W.W.); 15610565165@163.com (A.Z.); wzhe0427@163.com (Z.W.); tandaotong123@163.com (D.T.);
2. Key Laboratory of Marine Drugs of Ministry of Education, School of Medicine and Pharmacy, Ocean University of China, Qingdao 266003, China;
3. Infinitus (China) Company Ltd., Guangzhou 510405, China; Qingtao.yu@infinitus-int.com (Q.Y.); Kyle.yang@infinitus-int.com (K.Y.);
4. Laboratory for Marine Drugs and Bioproducts, Qingdao Marine Science and Technology Center, Qingdao 266237, China;

* Correspondence: caic@ouc.edu.cn (C.C.); glyu@ouc.edu.cn (G.Y.); Tel.: +86-532-8203-1609 (G.Y.)

† These authors contributed equally to this work.

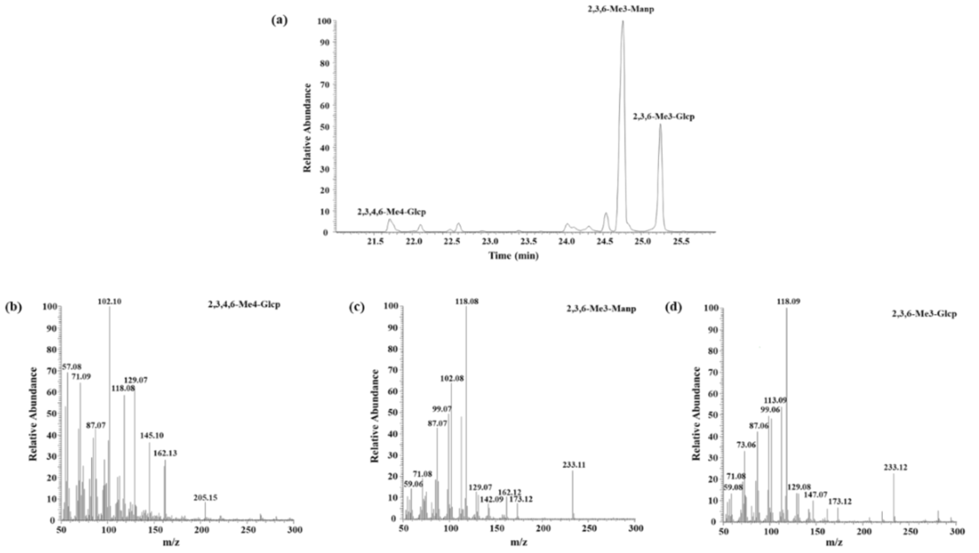

Figure S1. The GC-MS data of the partially methylated alditol acetates of FDOP-1A. (a) GC-MS elution spectra of FDOP-1A after methylation. (b) Mass spectrometry fragments of Manp-(1-. (c) Mass spectrometry fragments of -4)-Manp-(1-. (d) Mass spectrometry fragments of -4)-Glcp-(1-.

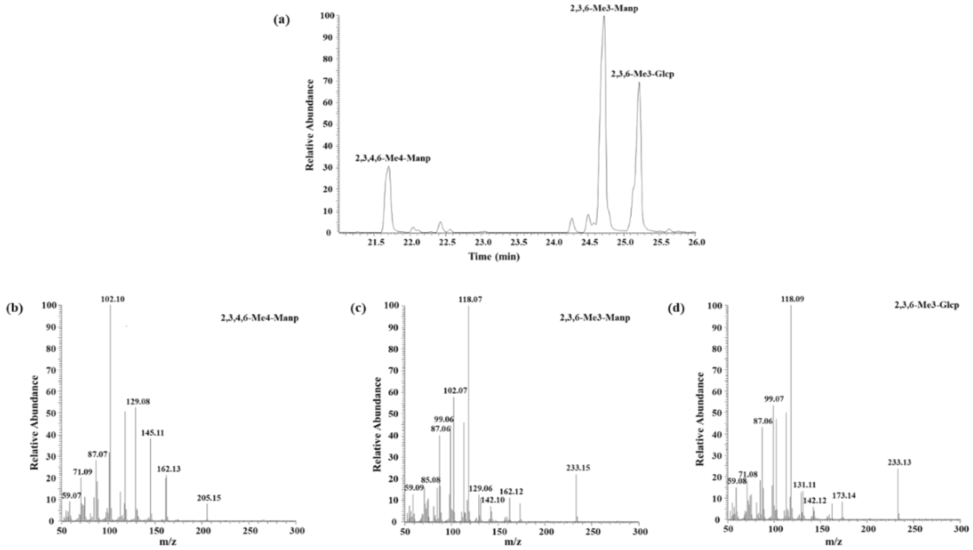

Figure S2. The GC-MS data of the partially methylated alditol acetates of FDOP-2A. (a) GC-MS elution spectra of FDOP-2A after methylation. (b) Mass spectrometry fragments of Manp-(1-. (c) Mass spectrometry fragments of -4)-Manp-(1-. (d) Mass spectrometry fragments of -4)-Glcp-(1-.

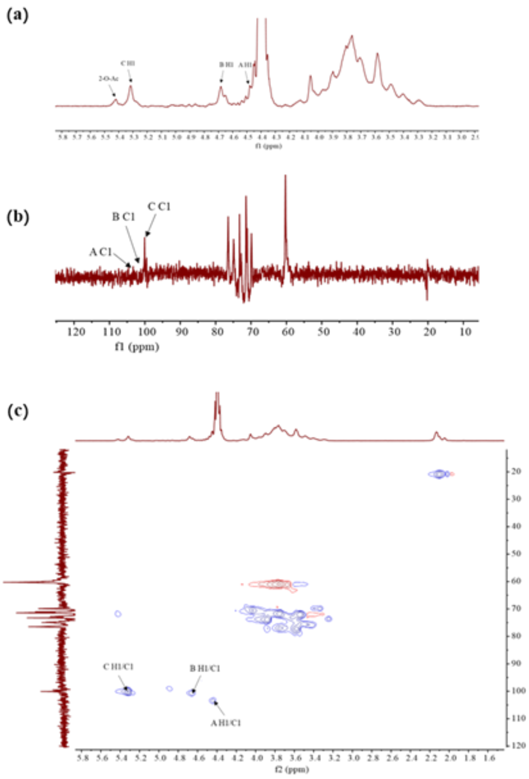

Figure S3. The NMR spectra of FDOP-2A: (a) ^{1}H-NMR spectrum of FDOP-2A in D_2O at 60 °C; (b) ^{13}C-NMR spectrum of FDOP-2A; (c) ^{1}H-^{13}C HSQC spectrum spectra of FDOP-2A.

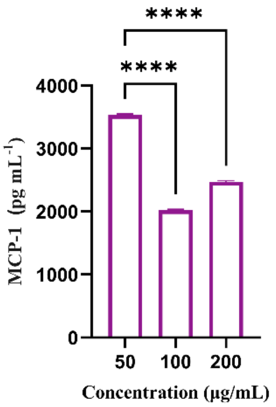

Figure S4. The effect of FDOP-1A on the production of MCP-1 in LPS-induced RAW 264.7 cells at concentrations of 50 μg/ml, 100 μg/ml, and 200 μg/ml.
